# Odontography; or a Treatise on the Comparative Anatomy of the Teeth; Their Physiological Relations, &c.

**Published:** 1846-01

**Authors:** 


### ART. IV.

1. *Odontography; or a Treatise on the Comparative Anatomy of the Teeth; their Physiological Relations, &c.* By RICHARD OWEN, F.R.S. &c. &c.—*London*, 1840-45. Royal 8vo, pp. 656. With 168 Plates.
2. *Report on the Microscopic Structure of Shells.* Part I. By WILLIAM B. CARPENTER, M.D. F.R.S. 8vo, pp. 24. With 20 Plates.
3. *Observations on the Structure of the Shells of Molluscous and Conchiferous Animals.* By JAMES SCOTT BOWERBANK, F.R.S. &c. Vol. I.—*London*, 1844. Royal 8vo, pp. 34. With 5 Plates.

IT will be in the recollection of our readers, that, in our last Number, we devoted a considerable space to the analysis of Prof. Owen's admirable researches on the comparative structure and development of the teeth; of which the publication is now complete. Our survey embraced a general account of the elementary types of Dental tissue; together with an outline of the principal forms which the apparatus presents, in the classes of fishes and reptiles. We now take up the subject where we left off; and, passing over the class of birds, whose total want of teeth leaves us nothing to say on the subject, we proceed with the dental apparatus of mammals; after which we shall give a brief notice of the recent discoveries made in a corresponding field of investigation, by Dr. Carpenter and Mr. Bowerbank.

MAMMALS. It is in the class of mammals, that the Dental apparatus presents its most special development, and is of the greatest importance in classification. This importance may be over-rated however; and has actually been, in Prof. Owen's opinion, by Frederick Cuvier. It is requisite to bear in mind, that there is a considerable diversity in the limits of variation, among different orders; thus the dental system of the *Cetacea* and *Bruta* has a much greater range of variation, and a less constant relation to the other characters on which the families and genera are founded, than in the ungulate and higher unguiculate groups.

"It is true, indeed, that the most manifestly natural mammalian genera are those, the species of which are provided with absolute similar molar teeth; and that those genera, which include species with molars of different forms, do not present the same character of unity. But it does not follow that, by combining species of mammals with similar molars, a group will be formed perfectly analogous to those which may be considered as the most natural or perfect. Neither the molar teeth, nor any other solitary character will serve to establish a natural classification.

"The molar teeth will least mislead in this respect, where their modification is most extreme, as when they are adapted to divide the flesh of animals, in which case they must of necessity be associated with the faculties and instruments for seizing and destroying prey. But molar teeth may be similarly modified, and equally well-adapted for crushing vegetable substances, which substances may be sought for by one species on the dry land, by a second in marshes, and by a third in the sea, or on the banks of rivers. The grinding surface of the molar tooth, for example, may for this purpose be elevated into a pair of transverse ridges; and we find such molars in the kangaroo, the tapir, and the manatee, as also in the extinct *diprotodon, nototherium,* and *dinotherium.* The small anterior molars of the *mastodon giganteus* likewise present this form. It would be difficult to select from the mammalian class the constituents of the more heterogenous group, than would be constituted by the character which M. F. Cuvier has assigned as the true guide in the formation of the most natural and uniform genera in mammalogy." (Introduction, p. lxxi.)

Even in regard to teeth adapted to carnivorous habits, if these characters were to form the sole guides in classification, species of placental mammalia would be associated with those of the ovo-viviparous sub-class. This was actually done by F. Cuvier; and other zoologists have followed in the same track with wonderful pertinacity. There is no limit to the absurdities which result from the adoption of any single character as the uniform guide in classification, however valuable such a character may generally be as an exponent of others. There is certainly no single character, which can be so much relied on in the arrangement of Mammals, as the conformation of the teeth; yet, as we have seen, the exclusive adoption of it leads to the most erroneous results. Next to the teeth, we may rank the extremities, as serving to afford important grounds of union or distinction; but to rely exclusively upon these, as is done by a British zoologist of some eminence, is to bring together the monkeys and opossums, because they have an opposable thumb, although in other respects they are nearly at the opposite extremities of the scale; and to reunite the carnivorous and herbivorous cetacea, which all truly scientific zoologists have agreed to separate, on account of their difference in all *essential* characters, their agreement being only in those which *adapt* them to the fish-like form and habits.

The class of Mammals, like the preceding, includes a few species which are entirely devoid of teeth, or of any representatives of those organs; these all belong to the group of anteaters, with the exception of the monotreme Echidna, which resembles the rest in regimen, though differing so remarkably in its generation. A few mammals have the jaws provided with horny substitutes for teeth, as the whalebone-whales and the ornithorhyncus. In the rest of the class, true teeth are present; these are always confined to the jaws; but the lingual and palatal teeth of reptiles and fishes are not without their representatives.

"In the feline tribe, the epithelium of the tongue is thickened at the fore part of its dorsum, and invests the papillæ there with hard sheaths like prickles, which are analogous to the lingual teeth of certain fishes and batrachians. The back part of the dorsum of the tongue in the echidna is provided with a plate of horny denticles, which bruise its food against the hard and prickly epithelium covering the palate. Horny processes, analogous to the palatal teeth of fishes and reptiles, are likewise developed upon the roof of the mouth of the great bottle-nose dolphin, thence termed *hyperoodon* by Lacépède." (p. 296.)

The average *number* of teeth in mammals, is that which characterizes man, the apes of the old world, and the true ruminants;—namely, 32. In some species of cetacea, however, this number is reduced to two; and in the narwhal or sea unicorn, only one is ordinarily developed, but this grows to an unusual length. The elephant has never more than one entire molar, or parts of two, in use on each side of the upper and lower jaws; making, therefore, at the most, eight molars in all; to which we have to add the two tusks, developed in the intermaxillary bones. The examples of excessive number of teeth are presented, in the order *Bruta*, by an extinct armadillo, which has 98 teeth; and among the *Cetacea*, by the cachalot or sperm-whale, which has upwards of 60 teeth, though most of them are confined to the lower jaw,—by the common porpoise, which has between 80 and 90 teeth,—by the gangetic dolphin, which has 120 teeth,—and by the true dolphins, which have from 100 to 190 teeth, yielding the maximum number in the class Mammalia. Where the teeth are in excessive number, as in the species just cited, they are, as among reptiles and fishes in similar circumstances, nearly alike in form; being for the most part simply conical, and evidently destined rather for the prehension than for the mastication of the food. In several of the bruta, again, such as the smaller armadillos, the orycterope, and the three-toed sloth, in which the anterior teeth are deficient, the remainder are nearly all of one form, being sub-cylindrical with broad triturating surfaces; they differ, however, in size. In almost all the other Mammalia, particular teeth have special forms for special uses; thus, the front teeth, from being commonly adapted to effect the first coarse division of the food, have been called cutters or incisors, and the back teeth which complete its comminution, grinders or molars; large conical teeth, situated behind the incisors, and adapted by being nearer the insertion of the biting muscles to act with greater force, are called holders, tearers, lamaries, or more commonly canine teeth, from being well developed in the dog and other carnivora, although they are given, likewise, to many vegetable-feeders, for defence or combat. These names are not, however, by any means indicative of the shape of the crowns of the teeth which occupy the respective positions just described; and it has been found necessary to consider the position, and also the mode of succession of the teeth, in order to make them of definite use in classification.

"Comparative anatomists, by common consent, now apply the term '*incisor*,' arbitrarily to those teeth which are implanted in the intermaxillary bones, and in the corresponding part of the lower jaw. When the tooth which succeeds the incisors, or the first of the upper maxillary bone, is conical, pointed, and longer than the rest, it is called a '*canine*,' as is also its analogue in the lower jaw, which always passes in front of it when the mouth is closed. Of the remaining teeth, those which are shed and replaced vertically, or which have successors descending into their place in the upper, and ascending in the lower jaw, are called '*premolars*,' or false molars, and in human anatomy '*bicuspides*;' the remaining teeth, which are not displaced by vertical successors, but which follow each other from behind forwards, in both jaws, are called '*molars*,' or true molars." (p. 298.)

The ordinary teeth of mammalia have so much more definite and complex a form than those of fishes and reptiles, that we can usually recognize in them the *crown* or exposed part, as not only distinct from the *fang* or inserted part, but as divided from it by a constricted portion or *neck*. In those teeth which grow uninterruptedly, however, the exposed part is not separated by a neck from the implanted part, which generally maintains to its extremity the same size and shape as the implanted crown. It is peculiar to the class Mammalia to have teeth implanted in sockets by two or more fangs; but this can only happen to the teeth of limited growth, and generally characterizes the molars and premolars. In no mammal does anchylosis of the teeth with the jaw constitute a normal mode of attachment, as in the cold-blooded vertebrata. Each tooth has its particular socket, to which it firmly adheres by the close co-adaptation of their opposed surfaces, and by the firm adhesion of the alveolar periosteum to the organized cement, which invests the fang or fangs of the tooth; but in some of the cetacea, at the posterior part of the dental series, the sockets are wide and shallow, and the teeth adhere more strongly to the gum than to the periosteum; so that Prof. Owen has seen, in the cachalot, all the teeth brought away with the ligamentous gum, when it has been stripped from the sockets of the lower jaw. True teeth implanted in sockets are confined, in this class, to the maxillary, intermaxillary, and lower maxillary bones, and to a single row in each. In general, teeth are situated in all these bones; but they may be absent in the intermaxillaries, as in the ruminants and most bruta; or they may be present in those bones only, as in the narwhal. In man, the intermaxillaries coalesce with the maxillary bones at an early period; it was formerly supposed that he was the only mammal in which there is no interspace in the dental series of either jaw,—a character resulting from the shortness of the jaws, and the equal lengths of the crowns of the teeth; but the importance of this character as associated with the peculiar attributes of human organization, has been somewhat diminished by Cuvier's discovery of a like contiguous arrangement of the teeth, in the jaws of the extinct *Anoplotherium.* For a general account of the *substance* of the teeth of mammals, we shall have recourse to Prof. Owen's unabridged description.

"The teeth of mammalia usually consist of hard or unvascular dentine, defended at the crown with an investment of enamel, and everywhere surrounded by a coat of cement. The coronal cement is of extreme tenuity in man, quadrumana, and terrestrial carnivora; it is thicker in herbivora, especially in the complex grinders of the elephant; and is thickest in the teeth of the sloths, magatheroids, morse, and cachalot. Vertical folds of enamel and cement penetrate the crown of the tooth in ruminants, and in most rodents, and pachyderms, characterizing by their various forms the genera of the two last orders; but these folds never converge from equidistant points of the circumference of the crown towards the centre. The teeth of the quadrupeds of the order bruta have no true enamel; this is absent likewise in the molars of the dugong, zeuglodon, and the cachalot. The tusks of the narwhal, walrus, elephant, mastodon, and dinotherium, consist of modified dentine, which in the large proboscidian animals is properly called 'ivory,' and is covered by cement.

"The central part of the fully-formed tooth in man and most other animals, contains an irregular kind of osseous substance, which is most abundant in the cachalot, and forms around foreign bodies which may gain admission to the pulp-cavity of tusks. A fifth substance which, from the number and regular position of the vascular canals in it, I have termed the 'vascular dentine,' forms the body or axis of the tooth in the sloth tribe, and is present in smaller proportion in the centre of the teeth of the armadillos. The teeth of the *orycteropus* consist of congeries of long and slender prismatic columnar denticles, each consisting of a body of dentine, with a coating of cement, by which they are united together to form a composite tooth, as in some of the cartilaginous fishes." (p. 302.)

"The dentine of the long and slender prismatic denticles, which are aggregated to form the compound tooth of the orycterope, is unvascular, and is characterized chiefly by the frequent division and wide angles at which the branches of the bifurcating calcigerous tubes diverge, before resolving themselves into their minute wavy terminations." (p. 303.)

The resemblance between one of the composite teeth of the mammiferous orycterope, as figured in plate 78 of the Odontography, and that of the saw-fish shown in plate 9, is very remarkable; and it shows what care is requisite in drawing general conclusions respecting the limitation of special forms of dental structure to particular groups, until the examination has been most extensively prosecuted.

As the account of the dental structures, which was contained in the preceding article, was principally founded upon the characters which they exhibit in mammalia, we need not stop here to go over the same ground again; but have only to add to our generalities a few words regarding the peculiarities of dental development in this class.

The primary pulp, which first appears as a papilla on the free surface of the gum, and by the calcification of which the dentine is formed, sinks into a cavity, and becomes surrounded by a closed capsule, at an early period of the formation of the tooth, in every mammal. It is also completely enclosed in the substance of the jaw-bone; from which the crown of the growing tooth extricates itself by exciting the absorbent process, whilst the cell is deepened by the same process and by the growth of the jaw, into an alveolus for the root of the tooth. In those teeth which possess enamel, the mould or pulp of that constituent is developed from the capsule covering the coronal part of the dentinal pulp. In the complex teeth of the herbivorous and some omnivorous quadrupeds, the enamel-pulp sends processes into depressions of the crown, which vary in depth, breadth, direction, and number, in the several groups; the dentinal pulp, thus penetrated, offers corresponding complications of form, and as the capsule follows the enamel-pulp in all its folds and processes, the external cavities or interspaces of the dentine become occupied by enamel or cement,—the cement, like the capsule which formed it, being the outermost substance, or having the enamel interposed between it and the dentine. The dental matrix presents the most extensive interdigitation of the dentinal and enamel pulps, in the capybara and elephant.

The ordinary process of the formation of the fang is, in its essential characters, the same with the continued production of the tooth which takes place in certain cases, as the tusks of the elephant, and the front teeth of the rodentia; the only difference being, that the pulp is reproduced in perpetually decreasing quantity in the former case; whilst it is regenerated below, as fast as it undergoes conversion above, in the latter.

"In the formation of a single fang, the activity of the reproductive process becomes enfeebled at the circumference, and is progressively contracted within narrower limits in relation to a single centre, until it ceases at the apex of the fang; which, though for a long time perforated for the admission of the vessels and nerves to the interior of the tooth, is in many cases finally closed by the ossification of the remaining part of the capsule. When a tooth is destined to be implanted by two or more fangs, the reproduction of the pulp is restricted to two or more parts of the base of the coronal portion, around the centre of which parts the sphere of its reproductive activity is progressively contracted. The intervening parts of the base of the coronal pulp adhere to the capsule, which is simultaneously calcified with them, covering those parts of the base of the crown of the tooth with a layer of cement. The ossification of the surrounding jaw being governed by the changes in the soft but highly-organized dental matrix, fills up the spaces unoccupied by the contracted and divided pulp, and affords, by its periosteum, a surface for the adhesion of the cement or ossified capsule, covering the completed part of the tooth." (p. 306.)

The following general statements in regard to the *succession* of teeth in the mammalia are extremely interesting. We have already noticed the discovery, first made we believe by Meckel, that the matrices of the permanent or secondary teeth of man are offsets, as it were, from those of the deciduous or primaries, whose places they take. The first permanent molar, however, originates from a primary matrix; this, it will be recollected, makes its appearance *behind* the two deciduous molars, which are replaced by the bicuspids. The second and third permanent molars, which come up successively behind the first, are offsets from it; or rather the second is a sort of bud from the first, and the third from the second, The following extract will show the relation between this history in man, and that which takes place in other mammalia:

"The matrix of certain teeth does not give rise, during any period of their formation, to the germ of a second tooth, destined to succeed the first; this, therefore, when completed and worn down, is not replaced: all the true cetacea are limited to this simple provision of teeth. In the armadillos, megatherioids, and sloths, the want of germinative power, as it may be called, in the matrix, is compensated by the uninterrupted growth of the teeth. In other mammalia the matrix of the first-developed tooth gives origin to the germ of a second tooth, which sometimes displaces, sometimes takes its place by the side of its predecessor and parent. All those teeth which are displaced by their progeny are called temporary, deciduous, or milk teeth: the mode and direction in which they are displaced and succeeded, viz. from above downwards in the upper, from below upwards in the lower jaw, in both jaws vertically, are similar to those in the crocodile; but the process is never repeated more than once in any mammiferous animal." (p. 307.)

The following extract relates to the cases in which the gemmiparous process allows the newly-formed teeth to come up by the side of their parents, as in the case of the second and third true molars of man:

"In this successive germ-production, we find repeated the multiparous property of the dental matrix of the crocodile; but the concomitant growth of the jaw allows the second, third, and sometimes even fourth generation of true molars to coexist, and come into place side by side. In the unguiculate and most of the ungulate species of the placental division of the mammalian class, the fissiparous reproduction of horizontally-succeeding teeth stops at the third generation; in other words, they have not more than three true molars on each side of the upper and under jaws. In the marsupial series the same process extends to a fourth generation of true or horizontally-succeeding molars; and in most of the species, the four true molars are in use and place at the same time; but in the kangaroos, the anterior ones are shed before the posterior ones are developed. This successive decadence is still more characteristic of the grinding-teeth of the elephant, which consist exclusively of true molars." (p. 308.)

We are rather surprised that Prof. Owen should not have included, amongst his general remarks on the teeth of mammalia, some notice of the curious arrest of development, which accounts for the absence of teeth in many species; but we may partly supply the deficiency by extracts from the subsequent detailed account of the dental apparatus in the several families of mammalia:

"The great whales, before they acquire their peculiar array of baleen plates, manifest in their fœtal age a transitory condition, a true dental system, which, though abortive and functionless, beautifully typifies that which is normal and persistent in the majority of the order. In an open groove, which extends along the alveolar border of both the upper and the lower jaws, there is a series of minute, conical, acute, or obtuse denticles, with hollow bases inclosing the uncalcified remains of a vascular pulp. These were first noticed by the philosophical anatomist, Geoffroy St. Hilaire, and have subsequently been described and figured by Prof. Eschricht. The subject examined by the latter author was the fœtus of a balænoptera, the jaws of which were about four inches in length. The inclosed alveolar groove of the upper jaw contained twenty-eight denticles, that of the lower jaw forty-two. The anterior denticles in both jaws were the smallest; but they increase in size more gradually, and maintain a greater regularity of form, in the lower jaw, where they are most numerous, and in which the typical dentition of the carnivorous cetaceans first manifests its plenary development in the great cachalot. In the upper jaw of the fœtal whale some of the denticles are double, two adhering side by side; and they offer in their varying extent of confluence no unapt resemblance to the stages of the fissiparous multiplication of an infusorial monad. There can be little doubt, indeed, that these literally 'double-teeth' have resulted from a spontaneous fission of the primordial pulp-cell, the divisions of which growing to a certain size, have again coalesced in the progress of their calcification, like the two primitively detached summits of the ornithorhyncus's grinder. We cannot avoid recognizing in these bicuspid denticles the representatives of the gigantic extinct cetacean, called *zeuglodon;* and they also call to mind the similarly-shaped ultimate molar in the dugong.

"These small teeth and their matrices entirely disappear before birth; yet the fetal whale comparatively long retains and palpably exemplifies the earliest stage of dental development in the higher mammals, viz., the open fissure, which in these is so rapidly closed, especially in the human subject. But beyond this stage the true dentition of the balænidæ is not destined to proceed; and they thus manifest, agreeably with the general laws of unity of organization, their closest relations to the typical characters of their order at the early periods of development, divesting themselves of part of the more general type, in order to assume their special and distinctive characters as they advance towards maturity." (p. 347.)

A similar development of tooth-germs which are never to attain maturity, presents itself in the ruminants, in which it was first detected by Mr. John Goodsir in 1839. The number of *germs* of deciduous teeth in this group is in all 32; namely, *six* molars, *two* canines, and *eight* molars, in *each* jaw. But the number of teeth which actually appear is usually only 20; namely, *six* incisors, *two* canines, and *six* molars in the *lower* jaw; and *six* molars, without either incisors or canines, in the upper. The rudiments of the deficient incisors and canines of the upper jaw were discovered by Mr. Goodsir in the embryos of the cow and sheep; and those of the deficient molar have been detected by Prof. Owen, very near the canines, in the fœtus of a fallow-deer. These rudiments do not usually attain to that stage of calcification which distinguishes the rudimental hidden teeth of the whales; but some of them go on to their full evolution, in certain aberrant forms of the order. Thus in the camel tribe, the third or outermost of the upper incisors, together with the canine and the first premolar, are completely calcified, and have permanent successors; and in the musk-deer and the muntjac the permanent canine attains unusual size, and becomes a formidable weapon; whilst the primitive rudiments of all their upper incisors and first molars disappear, without giving origin to any successors.

We do not find any notice of similar conditions in the early condition of that group of animals, which was designated by Linnæus as *bruta,* and which has received from Cuvier the less appropriate name of *edentata.* Comparatively few of them are truly *edentate,* or destitute of teeth; whilst, on the other hand, only two or three species have any teeth in the intermaxillary bone. It would be very interesting to know if the general rule of the presence of the germs, even where teeth are not developed from them, would hold good in this instance also; but opportunities for obtaining the necessary specimens, in the right stage of their development, are not of frequent occurrence.

It is in vain for us to think of following our Author, even in the most cursory manner, through the detailed description of the dental apparatus in mammalia, which occupies more than half of the entire work; and we must content ourselves, as we have been obliged to do in regard to the two previous classes, with adducing one or two remarkable examples in illustration of the value of his researches, and of the generalizations which he founds upon them. We alluded in our former notice to the important assistance which had been given by Prof. Owen's microscopical analysis of the structure of the teeth, in the determination of the marsupial nature of the Stonesfield fossil; and to the new light it had thrown on the character of certain remains, which had been appealed to in support of the reptile affinities of that fossil. Those remains, first described as saurian by Dr. Harlan, were at once referred by Prof. Owen to the mammalian type, on account of the implantation of the teeth by double fangs in deep sockets; the microscopic analysis of the teeth confirmed this view, and indicated the cetacean affinities of the fossil, which was at that time only known by fragments of the jaws; and the subsequent discovery of a nearly complete skeleton has demonstrated the truth of Prof. Owen's deductions. The name of *zeuglodon* has been given to this animal from the peculiarity in the form of the posterior teeth, which are formed of two lobes united by a narrow isthmus, so that a transverse section presents somewhat the form of an hour-glass. In Prof. Owen's opinion, the pulp was originally simple, and became subsequently divided into two parts by a sort of fissiparous reproduction, but the complete separation never took place, as it does in the cetacea usually. After the division of the pulp has taken place, its calcification proceeds by two distinct centres, which are each separately surrounded by concentric striæ of growth. From the other remains of this animal which have been discovered since the examination of its teeth by Prof. Owen, the cetacean nature of the animal is fully demonstrated; and its place is shown to be at the extremity of the series of carnivorous whales, approaching the dugong and other apodal pachyderms. Its length must have been near seventy feet. Here then a case, which seemed to call in question Prof. Owen's generalization respecting the mammalian nature of all two-fanged teeth, implanted in a distinct socket, was found, when fully investigated, to afford a powerful support to it.

The structure of the teeth in the various recent and fossil species of the order *bruta,* affords many points of great interest, of which we may stop to notice a few. We have already alluded to the remarkable organization of the teeth in the orycterope, or Cape anteater, certain peculiarities of which did not escape the notice of Fred. Cuvier. These teeth, which are in reality made up by the union of several distinct denticles, are continued solid and of the same dimensions to the bottom of their sockets, where they terminate by a truncated and undivided base, appearing as if destitute of a pulp-cavity at every period of its growth. This anomaly, however, is more apparent than real; for though the tooth as a whole has no such cavity, each of the component denticles has its base excavated by a conical pulp-cavity, as in other animals; and this pulp is persistent, as in the rest of the order bruta. The wide inferior apertures of these cavities constitute the pores observable on the base of the compound tooth of the orycterope, and give to that part a close resemblance to the section of a cane. The vascular pulp becomes ossified near the grinding surface of the tooth, and consequently a transverse section taken from this part presents the centres of the radiation of the calcigerous tubes filled up with bone. The base of each pulp is surrounded by a delicate vascular capsule, which becomes ossified about a line above the base, and forms the cementing substance of the congeries of denticles. Hence :

"The teeth of the orycteropus offer, when rightly understood, no anomaly or exceptional condition in their mode of development. Each denticle is developed according to the same laws, and by as simple a matrix as those large teeth in other mammalia, which consist only of dentine and cement. The dentine is formed by the centripetal calcification of the pulp, and the cement by the ossification of the capsule. Both pulp and capsule continue to be reproduced at the bottom of the alveolus, *pari passu* with the attrition of the exposed crown; and the mode and time of growth being alike in each denticle, the whole compound tooth is maintained during the life of the animal. The augmentation in the size of the whole tooth, during the growth of the jaw, is effected by the development of new denticles, and by a slight increase of size in the old ones at the base of the growing tooth, which in the progress of attrition and growth, becomes its grinding surface." (p. 320.)

We alluded in our former notice to the remarkable fossil genus *glyptodon,* whose extraordinary tesselated armour was long supposed, by many zoologists, to appertain to the gigantic *megatherium.* The two animals are now known, however, chiefly through the discoveries of Dr. Lünd and Mr. Darwin in South America, and Prof. Owen's investigations at home, to be quite distinct; the former being referrible, as might have been supposed from its armour, to the armadillo group, and the latter being the representative of the extraordinary family of *gravigrades,* of which the existing sloths are diminutive arboreal forms. Many of our readers, we hope, have seen and admired the gigantic cuirass of the glyptodon, which was set up a few years since in the Hunterian Museum, after the expenditure of a vast amount of time and ingenuity in the recomposition of the scattered elements. This extraordinary beast seems to have surpassed the rhinoceros in size ; but in its dentition it was adapted to a more exclusively vegetable diet than its allies among the armadillos, which are somewhat omnivorous. Remains of at least six species, differing most characteristically in their *tails,* have been recently added to the British Museum, and will shortly, we hope, be displayed to the public. The teeth of the armadillo tribe are harder than those of any other species of the order *bruta;* but, as in all that order provided with teeth, they are wholly devoid of cement. They consist, in both the existing and extinct armadillos, of three distinct substances, of which the unvascular dentine is present in greatest proportion, and forms the main body of the tooth; but it includes a small central axis of vascular dentine, and is surrounded by an extremely thin coating of cement. The calcigerous tubes of the dentine are continued from the vascular or Haversian canals of the central axis; many of them appear to anastomose, either directly or by intermediate cells, close to the cement; others are directly continued into that substance, and terminate in its cells. The canals of the central axis are few in number, have a regular or parallel arrangement, and do not anastomose by loops; some of them are continued along with short processes of the osseous substance a little way into the dentine; and a rich brush or pencil of calcigerous tubes radiates in strong irregular curves from the obtuse ends of these processes, before falling into the common parallel course. The following are the differences presented in the structure of the tooth of the glyptodon:

"The vascular osseous texture occupies a larger proportion of the centre of the tooth than in the small armadillos; it is harder than the dentine or cement, and rises upon the grinding surface in the form of a ridge, extending along the middle of the long axis of that surface, and in three shorter ridges at right angles to the preceding, at the middle of each of the three rhomboidal divisions of the tooth. The medullary canals are surrounded by fine compact concentric strata, but are wider than in true bone, and the calcigerous cells are fewer and less conspicuous: the canals bend towards the dentine, but without any regular or parallel arrangement. The calcigerous tubes assume their parallel and regular course sooner than in the armadillos; they have the same relative diameter, arrangement, and terminal ramification. The outer coat of cement, though not exceeding 1-10th of a line in thickness, is relatively thicker than in the armadillos: a large proportion of the terminal branches of the calcigerous tubes is continued into it, and these branches anastomose with the plexiform tubes which surround the calcigerous cells.

"Although the teeth of the largest of the extinct loricated bruta present so much more complicated a form than do those of the small existing species, their intimate texture and composition are essentially the same, and very distinct from what has been described in the orycteropus, and will subsequently be shown to characterize the dentition of the family of the sloths. Nevertheless the modified proportions of the constituent textures of the molars of the glyptodon, which produce the inequality in the grinding-surface of the crown, coincide with the more complicated form of that surface in adapting the tooth for a more strictly vegetable diet, than is indicated by the more simple molars of the existing armadillos." (p.325)

It is interesting to find this inference confirmed, by the presence in the glyptodon of a descending process of the malar bone, which in the sloths affords the masseter muscle a more extensive and favorable attachment for producing those horizontal triturating movements of the lower jaw, which are necessary for the comminution of their food,—consisting as this does of the leaves and young shoots of trees. If we restrict our survey of the dental system to existing mammals, that of the sloths appears anomalous, both as respects the number, the form, and the intimate composition of the teeth. But when their type of dentition is viewed in connexion with that of the extinct bruta of the megatheroid family, the repetition of all its essential characters, under various minor modifications, in the already known species and genera (to which number additions are being continually made) of those once numerous and gigantic quadrupeds, gives it as important a place in the classification of the mammalian teeth, as that of the dentition of the proboscidian pachyderms or of the ruminants. These phyllophagous *bruta,* both recent and extinct, are characterized by the following remarkable dentition: the teeth are implanted in the maxillaries alone, never in the intermaxillary bones; they are few in number, not exceeding five on each side of the upper jaw, and four in the lower, making sixteen in all; they are mainly composed of vascular dentine, having a thin investment of hard or unvascular dentine and a thick outer coating of cement; and, in common with the teeth of the other bruta, they grow from persistent pulps, and are implanted by a simple deeply-excavated base, not separated by a cervix from the exposed summit or crown. The intimate structure of the vascular dentine resembles that of the entire tooth of psammodus, ptycodus, and some other cartilaginous fishes; it is perforated by vascular or medullary canals from 1-600th to 1-1000th of an inch in diameter, with intervals of twice or thrice that breadth; and these canals proceed in a slightly undulating and sub-parallel course, from the internal surface connected with the pulp, to the hard dentine, at right angles for the most part to the plane of the pulp-surface, and obliquely to that of the hard dentine,—those vascular canals which proceed from the summit of the pulp being parallel or nearly so with the axis of the tooth, whilst those from the base of the pulp are transverse to the same axis, and the intermediate canals have an intermediate course. Processes of the vascular pulp are continued along these canals; consisting essentially of tracts of the primitive pulp which have remained uncalcified. The medullary canals are usually cylindrical, but not of equal diameter throughout; sometimes they present alternate expansions and constrictions; sometimes they bifurcate; and more rarely they anastomose by loops, the convexity of which is turned towards the hard dentine. They everywhere give off minute calcigerous tubes, which have a wavy course, and the finest branches of which terminate in the minute cells of the intertubular tissue. The hollow cylinder or thin layer of hard dentine, resembles very closely that of the human tooth in structure. At the first formation of the tooth, this substance covers the crown as well as the sides of the tooth; but the coronal portion, although originally nearly three times the thickess of the cylindrical or parietal portion, is worn away almost before the sloth reaches maturity, and is never reproduced. The depressed centre of the grinding surface is then formed by the vascular dentine only; that surface having its inequality maintained by the edge of the cylinder of hard dentine, in place of enamel, and being thus adapted to the comminution of the softer vegetable substances, as the leaves, buds, and tender shoots of trees. The cemental sheath presents some interesting peculiarities. The number of radiating cells which it contains, is very large; and the system of tubes with which they are everywhere connected, is very rich and minute. The principal trunks of this system resemble the calcigerous tubes of the dentine in size and arrangement; presenting, in many parts of the cement, a parallel course, nearly at right angles with its surface, especially where it is in contact with the dentine; and communicating freely with the dentinal tubes, as well as, by their fine subdivisions, with the calcigerous cells. The substance, which is thus evidently intermediate between the dentine and ordinary cement, is harder than the vascular dentine, but softer than the unvascular dentine; and it wears away the bevelled edge around the latter, so as to give it support, but not to interfere with the projection of its margin.

The following passage concisely expresses the characters of the enormou extinct congeners of the existing sloths:

"The larger leaf-devouring species of the order bruta, being too bulky to obtain their food by climbing, were compensated by such colossal proportions and Herculean strength, as to enable them to uproot and prostrate the trees on which they feed; certain toes on each foot were modified for support and progression of the body by hoofs on level ground; all the known species had complete clavicles, closed zygomatic arches, and a powerful tail as long as the hind legs, ancillary to the support of the enormous hinder parts. Every member of this most extraordinary family of quadrupeds, which was peculiar to the American continents, is now extinct; their teeth, which governed their diet, closely resembled those of their nearest existing congeners, the sloths." (p. 333.)

The general plan of the dental structure was in fact precisely the same, in these gigantic megatheroids, as in the comparatively feeble sloths of the present time; and the nature of their food *could* not have been essentially different. In particular, it may be affirmed, that these teeth could not have served the purpose of grinding down hard roots, which has been supposed by some to have been their destination. The train of reasoning by which Prof. Owen was led to reconstruct, as it were, not only the animals, but their habits and mode of obtaining their food,—with a fidelity and consistency which carries conviction to every one who will follow him through the details,—we shall perhaps take another opportunity of bringing under the attention of our readers, in a notice of the Catalogue of the Fossil Mammalia and Aves contained in the Hunterian Museum, which has been recently published by the College of Surgeons. At present, we must restrict ourselves to an indication of the chief points, wherein their teeth differed from those of the existing sloths. Each molar tooth of the megatherium is excavated by an unusually extensive conical pulp-cavity; from the apex of which a fissure is continued to the middle concavity of the grinding surface of the tooth. The central axis of vascular dentine forms a large portion of the cylinder; this is surrounded by a thin layer of hard or unvascular dentine; and this, again, is coated by the cementum, which is of very great thickness on the anterior and posterior surfaces, but which is thin where it covers the outer or inner sides of the tooth. The medullary canals of the vascular dentine very commonly terminate close to the hard dentine, by anastomosing loops, the convexity of which is turned towards the origin of the tubes of the hard dentine; and occasionally, one of the medullary canals may be observed to extend across the hard dentine, and to anastomose with a corresponding canal in the cement. The interspaces of these medullary canals are principally occupied by calcigerous tubes, which have an irregular course, form reticulate anastomoses, and terminate in very minute cells, at least one hundred times smaller than the calcigerous radiated cells of the cement. The tubuli of the hard dentine, which are given off from the convexity of the terminal loops of the medullary canals, are more regular in their arrangement; as they run in a parallel course from one surface of the layer to the other, at right angles with the axis of the tooth. As they approach the cement, they divide and subdivide, and become more wavy and irregular; their terminal branches take on a bent direction, and form anastomoses, dilate into small cells, and many are seen to become continuous with the radiating tubes of the contiguous cement. This last-named substance, which enters so largely into the composition of the grinders of the megatherium, is characterized in that extinct animal by the size, number, and regularity of the vascular or medullary canals which traverse it. Commencing at the outer surface of the cement, they run in a direction slightly inclined from the transverse axis towards the crown of the tooth, for the most part parallel to each other; in this course they divide a few times dichotomously, and finally anastomose in loops, the convexity of which is directed towards the layer of hard dentine, and in most cases is in contiguity with it. Fine calcigerous tubes are sent off, generally at right angles, from the medullary canals; these quickly divide and sub-divide, form anastomosing reticulations, and communicate freely with the similar tubes that radiate from the calcigerous cells. The calcigerous tubes that radiate from the cells nearest the hard dentine, and from the terminal loops of the vascular canals, intercommunicate freely with the calcigerous tubes of the hard dentine. There is thus a very strong analogy in *structure* between the vascular dentine of the megatherium, and its cementum; for both have vascular or medullary canals (representing the haversian canals of bone), calcigerous cells, and a system of tubuli connecting these with each other and with the vascular canals. But their *origin* is different; the one being formed by *centripetal* calcification of the dentinal pulp, whilst the other originates in the *centrifugal* calcification of the capsule. The direction of the continued nutriment, also, is opposite; but there is an evident facility, in the tooth of the megatherium, for the passage of nutrient fluid from each of these substances towards the other, not only by the anastomosis of their tubuli through those of the hard dentine, but by the inter-communication of their vascular canals.

"In the structure which the fossil teeth of the megatherium and its extinct congeners clearly demonstrate, we have striking evidence of their rich organization, and that they were once pervaded by vital activity. All the constituents of the blood freely circulated through the vascular dentine and the cement; and the vessels of each substance intercommunicated by a few canals continued across the hard or unvascular dentine. With respect to those minuter tubes, the more important as being more immediately engaged in nutrition, which pervade every part of the tooth, characterizing by their difference of length and course the three constituent substances, and which are derived, like the hypothetical 'vasa lymphatica' of the old physiologists, from the ultimate blood-vessels, they form one continuous and freely intercommunicating sytem of strengthening and reparative vessels, by which the plasma of the tooth was distributed throughout the entire tooth for its nutrition and maintenance in a healthy state.

"The grinding surface of the close-set molars of the megatherium differs, on account of the greater thickness of the cement on their anterior and posterior surfaces, from those of all the smaller megatheroids, in presenting two transverse ridges; one of the sloping sides of each ridge being formed by the cement, the other by the vascular dentine; whilst the unvascular dentine, as the hardest constituent, forms the summit of the ridge, like the plate of enamel between the dentine and cement in the elephant's grinder. The great length of the teeth and concomitant depth of the jaws, the close-set series of the teeth, and the narrow palate, are also strong features of resemblance between the megatherium and elephant, in their dental and maxillary organization. In both these gigantic phyllophagous quadrupeds, provision has likewise been made for the maintenance of the grinding machinery in an effective state; but the fertility of the creative resources is well displayed by the different modes in which this provision has been effected; in the elephant, it is by the formation of new teeth to supply the place of the old when worn out; in the megatherium by the constant repair of the teeth in use, to the base of which new matter is added, in proportion as the old is worn away from the crown. Thus, the extinct megatherioids had both the same structure and mode of growth and renovation of their teeth, as are manifested in the present day by the diminutive sloths." (p. 345.)

Our limited space forbids our entering any further into the elaborate details of this truly splendid work; although there are many points which we should gladly have brought under the notice of our readers; especially the comparison of the teeth of man with those of the lower tribes,—the carnivora in particular. By a long and patient study of mammalian dentition, Prof. Owen has been led to adopt views on this subject, which differ from those of the Cuviers and of Blainville, and which lead to an alteration in the *dental formulæ* which are usually received upon their authority. The characters upon which *their* classification of molar teeth is founded, are derived from their "form, size, and relative position;" whilst Prof. Owen, adopting the principle of "development" as his guide, has arrived at results which appear to us much more consistent and unquestionable. This is only one out of the many examples we might cite, of the philosophic character, which pervades the whole of this treatise, and which gives it a value far greater than it would acquire from the collection of details of which it is chiefly made up,—important as these are in themselves and their applications, and lucidly as they are arranged and described. But we trust that we have said enough to indicate the value which we set upon the 'Odontography,' and to give our readers some conception of its surpassing merits. Although its price may unfortunately place it beyond the reach of a large proportion of the members of our profession, and although the mass of details which it includes is such as to constitute a larger demand upon their time and attention than they can afford, yet we trust that no medical library will long remain without a copy of it, as a standard work of reference, we venture to say, not only for the present, but for all future generations, as long as anatomy and zoology shall be cultivated as sciences.

The thread of inquiry, which has become, in the hands of Prof. Owen, so valuable a clue to the mysteries of Nature, has been taken up and followed by other investigators, with distinguished success. The structure of the skeletons of invertebrated animals has been recently made the subject of minute observation by various microscopists; and the result has been precisely what might have been anticipated on *a priori* grounds.

Thus in Dr. Carpenter's 'Principles of General and Comparative Physiology,' published in October, 1841, the following passage occurs:

"The dense calcareous shells of the Mollusca, and the thinner-jointed envelopes of the crustacea, have been commonly regarded as mere exudations of stony matter, mixed with an animal glue secreted by the membrane which answers to the true skin. The hard axes and sheaths of the polypifera, however, have been also regarded in the same light; and yet, as will hereafter appear, these are unquestionably formed by the consolidation of what was once living tissue. From the analogy which the shells of mollusca and crustacea bear to the epidemic appendages of higher animals, there would seem reason to believe that the former, like the latter, have their origin in cells; and that these are afterwards hardened by the deposition of earthy matter in their interior" (§ 44.)

Acting upon this view, Dr. Carpenter commenced, in the spring of 1842, a series of inquiries into the structure of the shells of mollusca, crustacea, and echinodermata; which he has since continued to prosecute on an extended scale, embracing several hundred species, recent and fossil, in his investigations. About the same period, Mr. Bowerbank, who had previously communicated a paper to the Royal Society on the structure of coral, commenced an independent series of observations on the structure of the shells of mollusca; which had reference, however, rather to the formation of shell, than to its microscopic characters when complete; and which have been limited, in consequence, to a comparatively small number of species. Finding that their paths of inquiry were so distinct, these gentlemen, in a spirit of mutual good feeling, which we should be glad to see more common among the votaries of science, agreed to prosecute them independently of each other; and the results of their rescarches were simultaneously communicated, on Dr. Carpenter's part to the Royal Society, and on Mr. Bowerbank's to the Microscopical Society, in January 1843. Since that time, the observations of Dr. Carpenter have been carried on under the encouragement of the British Association, and in part with assistance afforded by its funds; and the Report before us is the first part of a detailed account of the varieties in the structure of shell in the different families and genera of mollusca; of which the second, completing the bivalves, was presented at the recent meeting of the Association, but has not yet been published. Having early found a much greater difference in the intimate structure of different shells, than might have been supposed from their external aspect, Dr. Carpenter was naturally led to inquire whether, as in the case of the teeth, these peculiarities are characteristic of certain natural groups; and he soon arrived at numerous highly-interesting conclusions on this point. The following quotation will show on what an extent of evidence these conclusions rest. After mentioning that, during the preceding year, he had made little short of 1000 preparations of shell-structure—in addition to the hundreds previously in his possession—he thus continues:

"A considerable part of my labour has been directed to the determination of the questions: whether an uniform structure prevails through every part of the same shell, so that the structure of the whole shell may be predicated from that of a small portion of it,—and whether the same structure is found in different individuals of the same species, and among different species of the same genus. It is obvious that a settlement of these questions must be of great importance in the application of the microscope to the determination of fossil shells; and I think that I am now entitled to answer them with some degree of confidence. I have, in a considerable number of instances, submitted *every portion* of a shell to microscopic investigation, selecting such specimens as, from the peculiar characters of their structure, would serve as types to which to refer others; and I have invariably found that an uniform structure pervades the whole of each; so that the examination of but a very small fragment is sufficient to determine the structure of the entire shell. I feel equally certain with respect to the correspondence between the structure of different individuals of the same species; as I have never found any decided variation, although I have in some instances examined several specimens of one kind." (p. 2.)

This constancy of structure is precisely what might have been expected upon *a priori* grounds; but it was necessary to meet the objections of those, who were not prepared to admit the existence of any organic structure in shell; and who, considering it in the light of an inorganic exudation, would regard any variation in its microscopic characters as the result of accidental circumstances. We happen to know that a distinguished foreign geologist, who has lately been in this country, alleged most emphatically and repeatedly, that it was *impossible* that any organic structure could exist in shell or any similar formations; and when invited to an ocular demonstration of the fact, he would only reply—"The microscope frightens me." Such men, like the Lord of Clumber, seem to be living half a century too late.

In a subsequent part of his first Report, Dr. Carpenter gives the following statement of the general results of his researches into the minute structure of shell, in regard to their zoological applications:

"My inquiries, so far as they have yet proceeded, tend to establish this position, *that where a recognizable and constant diversity presents itself in the elementary structure of the shell, among different groups that diversity affords characters which are to a very high degree indicative of the natural affinities of those groups.* It is not always that peculiarities sufficiently distinctive present themselves, even between what are regarded zoologically as distinct families; but where a marked diversity *does* exist, I believe that it will always be indicative of the affinities of the animal. Thus the conformity in structure between all the shells of one natural family is usually so close, than any strongly-marked difference in a particular genus would make me hesitate in admitting it into the group. I think it well at once to premise, that the characters derived from the intimate structure of the shell are not likely to serve for the distinction of *species* from each other, and that they will not often distinguish *genera;* but for the separation of some *natural* families, I believe that they will furnish the best *single set of characters* that the naturalist possesses, especially among particular groups, in which the application of other characters is very uncertain. (p. 16.)

We shall now briefly notice the principal forms of shell-structure described by Dr. Carpenter and Mr. Bowerbank; between whom there is a very general agreement as to all the leading facts. The most remarkable is that designated by both of them as *prismatic cellular substance;* and we may most briefly describe it by saying, that it precisely resembles the enamel of teeth, upon a large scale. It consists of a series of elongated prismatic cells, the parietes of which adhere together so as to form a consistent tenacious membrane; and the surfaces of this membrane display the extremities of the prisms, which are rather irregularly hexagonal or pentagonal. The cells are filled with carbonate of lime, which is evidently secreted into them by their own inherent powers. When a layer of this substance is broken across, the fracture is evidently fibrous; the fibres passing from one surface to the other. When the animal matter has been removed by decomposition, the calcareous *casts* of the interior of the cells remain *in situ* until disturbed, still forming a continuous layer of prismatic fibres; but as these fibres are no longer held together by any connecting medium, they are at once separated when a portion of the layer is rubbed between the fingers. This condition is sometimes met with in recent shells; but more frequently in fossil. The prisms do not always pass continuously from the outer to the inner surface. They are more numerous and smaller on the exterior, and several of them terminate in points about the middle of the layer; their places being taken by the remaining prisms, which are continued inwards, and enlarge so as to remain in contact with each other. The prisms show the transverse striæ, which have been noticed as visible in the enamel-fibres. Mr. Bowerbank considers these striæ as constituting a system of vessels; but this view is objected to, on various grounds, by Dr. Carpenter, who gives the following account of them:

"By submitting the *cut edges* of the membranous wall of the prismatic cell (in a vertical section) to a high magnifying power, under favorable circumstances, I have been able to discover what I believe to be the real cause of the transverse striation in question. The membrane evidently projects inwards at those parts, not in consequence of being pushed inwards from without, but because *its own thickness* is there increased. This appearance corresponds well with the conclusion already drawn in regard to the *progressive* formation of each layer of shell; and I am much inclined to believe that each transverse marking indicates a distinct deposit. Whether, during the time when this succession of deposits was taking place, the prismatic cells grew at their bases, and these lines indicate the additions which were progressively made to the length of the cells; or whether the long prismatic cells, as we now find them, are made up by the coalescence of a number of layers of flat pavement-like epithelium-cells, placed one upon another, and the lines indicate their points of junction,—I do not feel warranted in affirming with certainty, as the question could be only rightly decided by examining the shell in the progress of its formation, which I have not yet had the opportunity of doing. I am much inclined, however, to adopt the latter view, which was suggested to me by Prof. Owen. The coalescence of cells linearly arranged, so as to form a single long cell or tube, is an occurrence with which animal and vegetable physiologists are alike familiar. The idea derives strength from the fact that I have occasionally met with a layer of prismatic cellular structure, of such extreme tenuity that it was almost impossible to separate it, lying between thicker layers of the same shell of *pinna*. The cells of this layer, instead of being elongated prisms, were flat and pavement-like, resembling the epithelium of serous membrane; and it was in such that I have found the cytoblasts most perfectly preserved. It is hardly to be supposed that this layer was produced by a distinct act of shell-formation, as it would not add in any appreciable degree to the size or solidity of the shell; and it seems probable that it was a supplemental portion which had not coalesced with the remainder of the layer, of which it should properly have formed a part." (p. 8.)

This prismatic cellular substance always forms the *exterior* layer of the shells in which it occurs; and there seems much reason to regard it in the light of a *calcified epithelium*. The following account is given by Dr. Carpenter of its development:

"Although the prismatic cellular structure has not yet been observed in actual process of formation, yet certain appearances which are occasionally met with in the marginal portions of its newest layers, throw great light upon its mode of growth, and indicate its strong resemblance to cartilage in this respect; for in these situations we find the cells neither in contact with each other, nor polygonal in form, but separated by a greater or less amount of intercellular substance, and presenting a rounded instead of an angular border. Upon looking still nearer the margin, the cells are seen to be yet smaller, and more separated by intercellular substance; and not unfrequently we lose all trace of distinct cells, the intercellular substance presenting itself alone, but containing cytoblasts scattered through it. This appearance has been noticed by myself in *perna* and *unio*, and by Mr. Bowerbank in *ostrea;* so that I have no doubt that it is general in this situation. We may, I think, conclude from it that the cells of the prismatic cellular structure are developed, like those of cartilage, in the midst of an intercellular substance, which at first separates them from each other; that as they grow and draw into themselves the carbonate of lime poured out from the subjacent surface, they approach each other more nearly; and that as they attain their full development, their sides press against each other, so that the cells acquire a polygonal form, and the intercellular substance disappears." (p. 9.)

Under the general head of *membranous shell substance,* Dr. Carpenter describes various forms of structure, which agree in not presenting distinct traces of a cellular origin, and which, in particular, leave only a *membranous* residuum when treated by acid. This membrane is evidently analogous to the "basement-membrane" of Mr. Bowman, the "primary membrane" of Mr. Goodsir; and the varieties in the appearances presented by it are thus described by Dr. Carpenter:

"In its simplest condition it is a pellucid structureless pellicle of extreme delicacy and transparency, exhibiting no trace either of cells, granules, or fibres. I have occasionally found it, however, of a somewhat granular appearance, as if formed by the solidification of a thin stratum of fluid, including an immense number of minute molecules. In other cases, again, I have found it studded here and there with what seemed to be incipient cells; and lastly, I have occasionally found these cells more developed, and forming an almost continuous layer on the surface of the membrane. In this state they somewhat resemble the incipient form of the prismatic cellular substance. These cells may be occasionally seen in sections of the shell itself; and they will be often found in very different degrees of development even in the corresponding layers of two shells of the same species. Coupling the appearances which I have myself observed with the observations of Mr. Bowerbank, on the *formation* of shell, and keeping in view the general doctrines of cell-action, which I have elsewhere endeavoured to develope, I am inclined to believe that these cells are, like the cells of the prismatic cellular structure, the real agents in the production of the shell, it being their office to secrete into their own cavities the carbonate of lime supplied by the fluids of the animal. But whilst the cells of the prismatic cellular structure advance in their development, so as to form a perfect tissue, the 'calcigerous cells' of which we are now speaking, appear to burst or liquefy, and to discharge their contents upon the surface of the subjacent membrane, on which a shelly layer is thus formed. A greater or smaller proportion of these being left entire, and being included in the substance discharged from the rest, would present the appearances I have mentioned as occasionally manifesting themselves in sections of membranous shell-structure, and in the decalcified membrane." (p. 10.)

In his second Report, Dr. Carpenter described a considerable group of bivalve shells constituting the family *myidæ,* in which the cellular character is very distinctly exhibited in sections of the unaltered shell, but which yet presents no cellular residuum; and he attributed this to the want of that horny intercellular substance which holds together the cells in the prismatic cellular structure, and which gives a degree of consistence to the animal basis that is altogether wanting elsewhere.

We must pass over Dr. Carpenter's observations upon the peculiar form of the membranous shell-substance which gives rise to the *nacreous* lustre, to extract his statements regarding the tubular form of structure, which evidently bears a close analogy to dentine :

"All the different forms of membranous shell-structure are occasionally traversed by tubes, which seem to commence from the inner surface of the shell, and to be distributed in its several layers. These tubes vary in size from about the 1-20,000th to the 1-2000th of an inch, but their general diameter in the shells in which they most abound is about 1-4500th of an inch. The direction and distribution of these tubes are extremely various in different shells ; in general, where they exist in considerable numbers, they form a network which spreads itself out in each layer, nearly parallel to its surface, so that a large part of it comes into focus at the same time, in a section which passes in the plane of the lamina. From this network some branches proceed towards the nearer side of the section, as if to join the network of another layer; whilst others dip downwards, as if for a similar purpose. . . . In no cases have I seen any such variation in the size of the tubes of the same shell, as would convey the idea of their resemblance to blood-vessels; and even where a division occurs, the size of each of the branches is usually equal to that of the single trunk. Sometimes these canals are quite straight, whilst in other instances they are sinuous. That they are not mere channels or excavations in the shell-substance, is proved by the fact that they may be seen in the decalcified membrane. I have frequently seen in them indications of a cellular origin, as if they had been formed by the coalescence of a number of cells arranged in a linear direction; and I find that Mr. Bowerbank has come to the same conclusion." (p. 14.)

The larger of these tubes are designated by Mr. Bowerbank as haversian canals ; but we scarcely see how they can deserve that title, as they are manifestly too small to contain a network of blood-vessels on their walls. The term might perhaps be more appropriately applied to certain perforations in the shells of the *terebratula* and allied genera, which pass almost directly through the shell, from surface to surface, and have a diameter sometimes amounting to about 1-400th of an inch. These are lined by a distinct tubular membrane ; and they are stated, in Dr. Carpenter's second Report, to be occupied by cells. They consequently bear a strong analogy to the medullary canals, described by Prof. Owen as existing in vascular dentine ; but no systems of radiating calcigerous tubuli have their origin in them.

A most remarkable analogy to unvascular dentine is found in the shell of the crab, and particularly in the end of the claw, where the calcareous envelope is thicker and of denser texture than in other parts. This is described by Dr. Carpenter (Annals of Natural History, Dec. 1843,) as "composed of a substance exactly analogous to ivory; being very transparent and apparently homogeneous when cut into very thin slices ; and being perforated by an immense number of minute sinuous tubuli, which run nearly parallel to one another from one surface of the shell to the other. A transverse section of the claw shows the tubes radiating from the central cavity towards the external surface, and would, I feel assured, be regarded by the most experienced observer as the section of a tooth, if he were not informed of its real nature."

The following is an illustration of the value of the microscopic examination of the intimate structure of shells, in determining the place of a specimen, whose position was otherwise doubtful. We should premise that all the shells of the family *margaritaciæ* agree with that of the *avicula* (or pearl-oyster,) in having a nacreous interior, and an exterior of prismatic *cellular* substance; whilst in the neighbouring family *pectinidæ*, (of which the common pectens or scallop-shells constitute the type,) the most characteristic feature is the coarsely-corrugated disposition of the *membranous* basis, and the occasional presence of tubuli. Now this corrugated structure, with a greater or less amount of tubular perforation, is characteristic of several other families of bivalve-shells, in which the mantle is wholly or partially closed, and which are therefore organized on a different plan from the pectenidæ, which have it open; and it would not therefore serve by itself to distinguish a fragment of a shell of this family from those alluded to. But it is *quite sufficient* to distinguish a shell of this family from any of the neighbouring families, to which in its general characters it might present an affinity; and it therefore affords the most assistance where it is most wanted, as in the following case:

"A shell was described by Prof. Philips, in his 'Geology of Yorkshire,' as an *avicula*, which had been previously described by Messrs. Young and Bird as a *pecten*. The same species, or one closely allied to it, found near Bristol, was described by Mr. S. Stutchbury as an avicula, he not being at the time aware that it had been met with and described elsewhere. The mixture of characters is such as would sanction its being placed in either group, according to the relative value attached to them. Thus in the form of its hinge, it is most allied to avicula; whilst in the flatness of its under valve, and in the disposition of its costæ, it rather corresponds with the pectens. The intimate structure of the shell here serves, I think, to decide the point; for we find no trace of either the prismatic cellular substance or the nacre, which are characteristic of avicula, but we meet, on the other hand, with the coarsely-corrugated and somewhat tubular structure of the pectenidæ." (p. 20.)

In the same manner, Dr. Carpenter has been able to give important assistance in the determination of the true affinities of the curious aberrant genus *etheria*, an inhabitant of the *rivers* of Africa, and yet so *oyster-like* in its aspect, that it gave rise to Bruce's statement that he had seen oysters above the cataracts of the Nile; which, like many other assertions of that enterprising traveller, was the subject of much ridicule at the time, but has been since shown to have a strong foundation in truth. By most conchologists it has been arranged among the *chamaceæ*, chiefly on account of its tendency to attach its lower valve to solid bodies; this tendency, however, exists equally among the true *oysters*, from which, in many points of its organization, as well as in its habitat, it seemed widely separated. A better knowledge of its anatomy, however, has shown that its place could not properly be among the chamaceæ, and that it should be referred to the margaritaceæ, or to one of the groups bordering upon it, and more nearly allied to the oyster; and this view exactly corresponds with the characters presented by the intimate structure of the shell, when examined by Dr. Carpenter, for it was found to consist of a prismatic cellular substance with a lining of nacre.

We must now quit Dr. Carpenter's Report, to give an extract from Mr. Bowerbank's 'Observations,' which will convey an idea of his results in regard to the subject to which they are chiefly directed:

"If the newly-formed lip of the common garden-snail be carefully removed, so as to include a portion of the shelly matter as well as the membrane, and be put into a little water, covered with a thin slip of glass, and examined by transmitted light with a power of 280 linear, it presents the appearance of a thin, yellow-coloured, horny membrane, thickest at its junction with the old lip of the shell, and becoming gradually thinner towards the outer margin. There are an infinite number of minute globular vesicles scattered over the whole of its surface, which vary in diameter from 1-50,000th to 1-3000th of an inch. These vesicular bodies are incipient cytoblasts and cells; for although in the early stage of their growth we cannot discern the nucleus, they may be readily traced through their various stages of development, until in the larger vesicles it may be frequently observed; and the young cells approaching each other are compressed closely together, and become the first superficial layer of cellnlar structure. . . . The completion of the process of the development of the cellular tissue may be best observed in the immediate neighbourhood of the old lips of the shell; and passing thence gradually towards the outer margin of the newly-formed membrane, they may be seen in all their different stages. While the young cells are detached from each other, and retain their globular forms, they are perfectly transparent, the secretion of calcareous or other matters not appearing to have commenced; but we frequently observe in the neighbourhood of the newly-formed stratum small patches of cells congregated together, which are of a deep yellow tinge, and of a semi-opaque appearance, as if they were separate centres of ossification, arising in the same manner that we observe in the production of the calcareous matter in the young cartilages of the bone in the higher classes of the animal kingdom. Amid the great mass of vesicular bodies destined to form the cells for the secretion of the calcareous deposits of the shell, we find other cytoblasts dispersed over all parts of the membrane, which are developed in the form of tesselated cellular structure, by which means the original membrane is very much increased in substance. This production of tesselated cellular structure is more particularly abundant when the calcigerous cells are in their advanced stages of development. If a portion of the newly-formed stratum of shell be removed from the membrane on which it reposes, we observe that the horny structure continues to abound in the vesicles in all their earlier stages of growth; from which it would appear that its office is not confined to the production of the first stratum of calcigerous cells only, but that it is destined to originate the greater part, if not the whole, of the calcigerous cells of the shell; and this appears the more probable, as we may now observe that the membrane has attained a higher stage of organization, minute vascular tissue being frequently observed imbedded aud ramifying amid its structure, and oftentimes projecting from its torn edges. These vessels are very minute, none of those which I have observed exceeding 1-14,000th of an inch in diameter. If the uncovered portion of the membrane nearest to the old lip of the shell be examined with a power of 500 linear, these minute vessels may be seen in the course of formation; presenting the appearance of long ramifying lines of exceedingly minute gelatinous molecules, closely adhering together in a single series, and frequently appearing cylindrical and tubular in one part, while in another they have quite a moniliform appearance. This production of vascular structure by the arrangement of minute molecules or cytoblasts in single series, I have frequently observed in the membranous tissues of the corallidæ." (p. 6.)

Thus it appears that the hard skeletons which afford support and protection to the soft bodies of animals belonging to the widely-different families among the invertebrated classes, are formed upon a plan essentially the same with that which may be traced in the production of the various kinds of dental structure in the vertebrata. This analogy might in some degree be predicated; when it is considered that the teeth are produced from the mucous membrane, and may be regarded as parts of the *dermo-skeleton,* which is developed to the exclusion of the internal or *neuro-skeleton,* in the lower divisions of the animal kingdom. We have no doubt that the careful study of the history of their development, pursued in connexion one with the other, will lead to some very interesting general results, or rather to an extension of those which have been already attained. Thus it appears from the observations of Mr. Bowerbank, that the tubuli in shell have precisely the same origin as in tooth, being the spaces originally occupied by nuclear particles arranged in rows, and therefore not filled up in the process of calcification. In the larger tubuli these particles are fully developed into cells, which Mr. Bowerbank states that he has frequently met with in cylindrical series; each cell having a distinct nucleus, a transverse diameter of 1-5000th of an inch, and a longitudinal diameter of from 1-3000th to 1-200th of an inch.

The following observation confirms Dr. Carpenter's views, founded upon the structure of the complete shell, in regard to the succession of deposits in each new formation:

"From a careful examination of many new lips in various stages of progressive development, I do not think that the whole of the coagulable lymph of which the new membrane is formed, is exuded from the surface of the animal at one operation, but rather by a series of exudations, each one of which emerges from beneath the terminal margin of the last one; for if the membrane be carefully examined from the line of advance of the newly-formed cells towards its extreme edge, it will be seen that there are several lines of thickened tissue, which correspond in distance and appearance with the lines of growth on the old shell; and this is the more probable, as I have observed in young shells which I have taken from their places of hybernation in November, that the extension of the lip is still going forward, or rather perhaps has been so up to the very period of their retirement for the season." (p. 7.)

We have given a less full account of the researches of Dr. Carpenter and Mr. Bowerbank than of those of Prof. Owen, not because they are less interesting in themselves, but because they have less bearing upon the chief objects of our Journal. None of our readers, we hope, will any longer retain any scepticism with respect to the value of the achromatic microscope as an instrument of research. It may almost be said to furnish the physiologist with a sixth sense, so greatly do its capabilities surpass those of the older instruments.

